# Innovative Materials for High-Performance Tin-Based Perovskite Solar Cells: A Review

**DOI:** 10.3390/polym16213053

**Published:** 2024-10-30

**Authors:** Xiansheng Wang, Jianjun Yang, Jian Zhong, Junsheng Yu, Xinjian Pan

**Affiliations:** 1 College of Electron and Information, Zhongshan Institute, University of Electronic Science and Technology of China, Zhongshan 528402, China; 2 School of Optoelectronic Science and Engineering, University of Electronic Science and Technology of China, Chengdu 610054, China

**Keywords:** self-assembling materials, material design, tin-based perovskite solar cells

## Abstract

With the rapid development of lead-based perovskite solar cells, tin-based perovskite solar cells are emerging as a non-toxic alternative. Material engineering has been an effective approach for the fabrication of efficient perovskite solar cells. This paper summarizes the novel materials used in tin-based perovskite solar cells over the past few years and analyzes the roles of various materials in tin-based devices. It is found that self-assembling materials and fullerene derivatives have shown remarkable performance in tin-based perovskite solar cells. Finally, this article discusses design strategies for new materials, providing constructive suggestions for the development of innovative materials in the future.

## 1. Introduction

**Figure 1:**
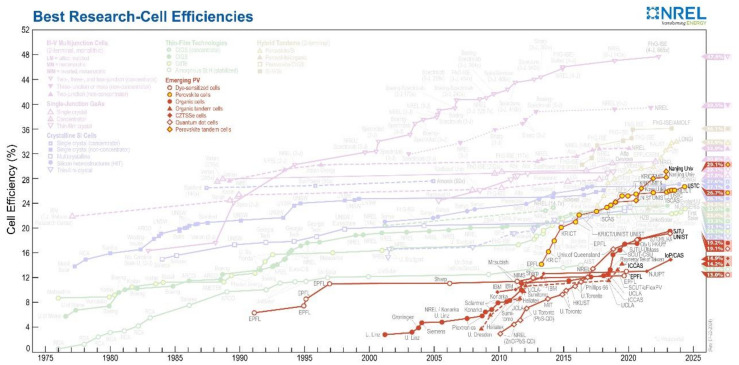
NREL announces latest perovskite solar cell efficiencies.

Solar energy is a promising renewable resource, especially perovskite solar cells (PSCs), which have rapidly advanced since Kojima et al. first proposed them in 2009 [1]. In recent years, they have reached a world-record power conversion efficiency (PCE) of 26.7% [2]. The efficiency development history of emerging photovoltaic cells is shown in Figure 1, where the efficiency of perovskite solar cells is represented by a yellow circular line graph. Organic–inorganic hybrid perovskites materials typically have the chemical formula ABX₃, where A is an organic or inorganic cation (such as methylammonium, formamidinium, cesium, or rubidium), B is a central metal cation (such as lead, tin, or germanium), and X is a halide or pseudohalide (such as chloride, bromide, iodide, or thiocyanate) [3]. Perovskite materials exhibit excellent optoelectronic properties, including tunable bandgaps [4], high absorption coefficients [5], ambipolar charge transport [6,7,8], defect tolerance [9,10], and low exciton binding energies [11,12]. Lead-based PSCs have achieved the best records in photovoltaic performance, but the toxicity of lead remains a major obstacle for industrialization, making the search for non-toxic or low-toxicity alternatives especially important [13,14,15,16,17].

Non-toxic candidates such as germanium, bismuth, and antimony have been proposed as substitutes for lead, but their photovoltaic performance falls far short of the 26% achieved by lead-based PSCs. Tin-based perovskites, as a low-toxicity alternative, have been extensively studied, and their PCE has reached over 15% [18]. Tin, being in the same IVA group as lead, has a similar ionic electronic configuration and comparable ionic radius [19,20,21], requiring only slight lattice distortion for substitution [22]. Although some concerns have been raised about the environmental impact of Sn^2+^ [23,24], it is generally believed that the degradation byproduct SnO₂ is non-toxic and easier to remove from the environment compared to the degradation products of lead-based perovskites. Therefore, tin-based perovskites show great potential for the development of high-efficiency solar cells, particularly in addressing the lead toxicity issue.

Although tin-based perovskites exhibit optoelectronic properties similar to or even superior to lead-based perovskites, such as higher carrier mobility and longer hot carrier lifetimes [25], their poor stability and the rapid oxidation of Sn^2+^ limit photovoltaic performance and reproducibility [26]. In practical fabrication processes, obtaining dense and pinhole-free films is also challenging. Tin-based perovskite films typically show poor crystallinity and rough surface morphology, with grain sizes in the range of hundreds of nanometers, unlike lead-based perovskite films that can achieve grain sizes in the range of hundreds of micrometers [27].

Review articles on tin-based PSCs have been published progressively. Among the classics, Loi focused on the structural characteristics and optoelectronic properties of 3D Sn-based perovskites, analyzing the impact of low-dimensional structures [28]. Yan et al. explored the fundamental properties of tin-based perovskites, device design, and various strategies to improve device performance [29]. Han reviewed the latest progress in efficiency, discussing the potential to enhance Sn-based PSCs’ efficiency by optimizing bandgaps, enhancing light-harvesting efficiency, increasing carrier diffusion lengths, performing surface passivation, and adjusting interfacial energy level alignment, pointing out opportunities and challenges in achieving 20% PCE [30]. Huang et al. examined the recent developments in inverted Sn-based device structures, emphasizing the characteristics, device performance, charge transport layers, and crystallization processes of tin-based perovskites, and explored future obstacles and opportunities [31]. Among all TPSC structures, the inverted structure is more commonly applied, while the conventional structure typically exhibits lower Voc and PCE. Due to intrinsic properties, tin-based perovskites’ electron diffusion length is longer than the hole diffusion length, making the inverted structure more suitable for TPSCs [32]. This paper mainly focuses on the polymer materials used in the tin-based perovskite solar cells, analyzes their characteristics, and provides suggestions for exploring novel modification materials. It is divided into three parts: polymer materials at the buried interface of perovskite films, polymer materials in the perovskite precursor solution, and polymer materials on top of perovskite films.

## 2. Application of Polymeric Materials in Tin-Based Chalcogenide Solar Cells

### 2.1. Polymer Materials at the Buried Interface of Perovskite Films

**Figure 2:**
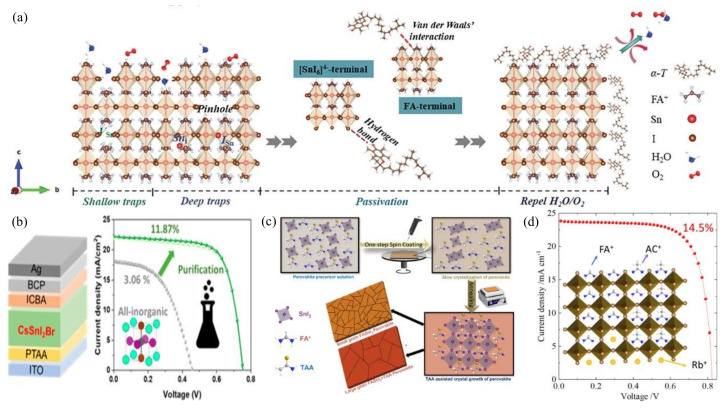
(**a**) Schematic illustration of defect profile in pristine Sn perovskite film with a variety of Sn- or I-related defects, and corresponding passivation strategy caused by hydrogen bonding and van der Waals interactions between perovskite and α-T [35]. (**b**) Efficient devices prepared with optimal Br content [33]. (**c**) Schematics of interactions and grain growth mechanism of FASnI3 perovskite with thioacetamide [36]. (**d**) Adding 10% AC and 3% Rb optimizes the device (E_1_AC_10_Rb_3_) to achieve 14.5% power conversion efficiency [37].

Up to now, the highest power conversion efficiency (PCE) of tin-based perovskite solar cells (PSCs) has exceeded 15%. However, this is still significantly lower than the theoretical limit. The main bottlenecks include a large open-circuit voltage (Voc) loss, uncontrollable crystallization dynamics, and poor air stability. One effective approach to address these challenges is through modifying the buried interface. This strategy not only precisely tunes the surface properties of the buried layer to enhance interfacial compatibility, but it also interacts with the perovskite layer, regulating the crystallization process or passivating the film [33,34,35,36] (Figure 2).

**Figure 3:**
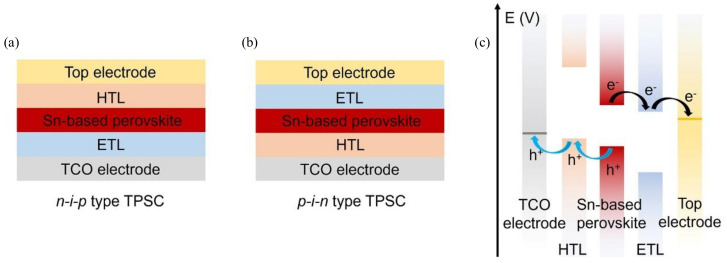
Schematic diagrams of (**a**) the n-i-p-type TPSC and (**b**) the p-i-n-type TPSC [38]. (**c**) Energy level diagram of a typical p-i-n-type TPSC [38].

In the study of buried interface modifications, the choice of the buried layer varies depending on the diversity of perovskites and device structures, whether conventional or inverted. For example, in lead-based PSCs, the n-i-p structure typically adopts TiO_2_/perovskite or SnO_2_/perovskite interfaces [37,38], while the p-i-n structure tends to use poly [bis(4-phenyl)(2,4,6-trimethylphenyl)amine] (PTAA)/perovskite or self-assembled monolayer (SAM)/perovskite interfaces [39,40] (Figure 3). In the case of tin-based PSCs, the mainstream choice is the poly(3,4-ethylenedioxythiophene)(styrene sulfonate) (PEDOT:PSS)/perovskite interface.

**Figure 4:**
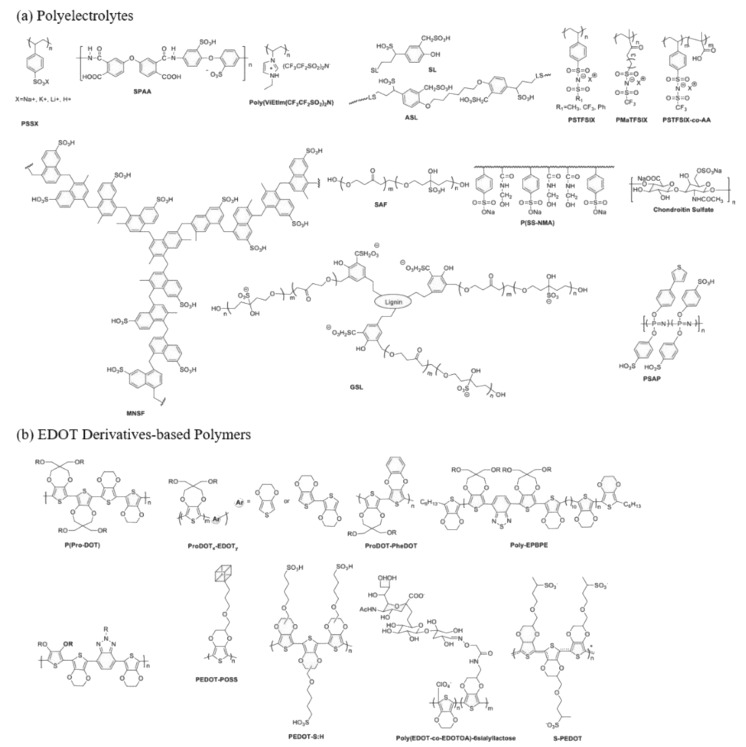
Schematics of representative (**a**) polyelectrolytes and (**b**) soluble processable polymers with EDOT derivates via copolymer engineering and side-chain tailoring [39].

PEDOT:PSS is a common choice due to its unique advantages such as high optical transparency, ease of solution processing, electrical conductivity, and energy level alignment with tin perovskites [39] (Figure 4). However, its acidic and hygroscopic nature can lead to unfavorable interactions with the tin perovskite interface, significantly affecting the performance of tin-based PSCs [41,42]. Studies have shown that the high defect density at the Sn perovskite/PEDOT:PSS interface induces severe non-radiative recombination and low charge carrier extraction/transport efficiency, resulting in significant reductions in both Voc and short-circuit current density (Jsc) [43,44]. Additionally, the PEDOT:PSS surface is often rich in PSS, whose insulating and hygroscopic nature further worsens carrier collection efficiency and accelerates degradation processes related to the buried interface [45].

**Figure 5:**
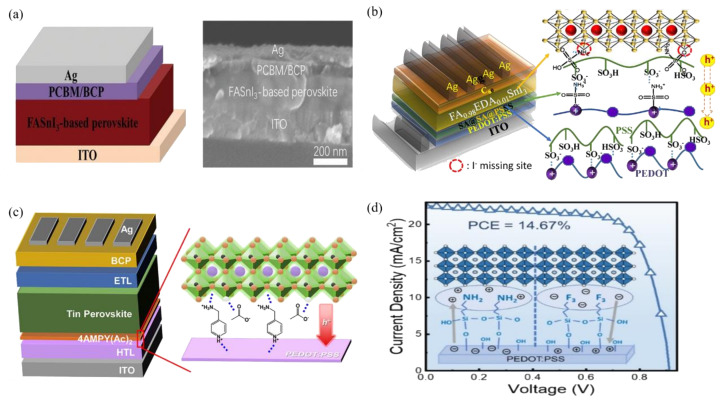
(**a**) Device configuration of the HTL-free tin PSCs with a structure of ITO/FASnI_3_-based perovskite/PCBM/BCP/Ag and SEM cross-sectional image of the HTL-free tin PSCs [48]. (**b**) A layer of PEDOT:PSS was inserted in-between the ITO electrode and SA-modified PEDOT:PSS to form a pseudo bilayered PS/SA@PS HTL [40]. (**c**) Post-treatment of PEDOT:PSS surfaces using diammonium salts of aromatic acetic acid dissolved in highly volatile but interacting solvents to modify the surface [45]. (**d**) Trimethoxy(3,3,3-trifluoropropyl)silane (F3-TMOS) with molecular dipole moments pointing to the hole transport layer was used as a buried interface modification material [34].

To address these issues, researchers have explored several alternatives to PEDOT:PSS, such as HTL-free materials, inorganic metal oxides, and novel p-type organic materials, significantly improving the stability of Sn-PSCs [46,47,48,49] (Figure 5a). However, compared to devices containing PEDOT:PSS, those without PEDOT:PSS often show poorer efficiency and reproducibility, likely due to the hydrophilic nature of PEDOT:PSS, which promotes crystal nucleation in the perovskite film. Another improvement strategy involves modifying PEDOT:PSS HTLs to enhance efficiency and stability [34,50,51]. For example, Song et al. successfully achieved a PCE of 12.1% and stability exceeding 2800 h in Sn-PSCs by post-treating the PEDOT:PSS surface with ethylenediamine [45] (Figure 5c). Further research has revealed that excess insulating PSS is partially removed during treatment, and the molecular structure of PEDOT:PSS shifts from benzenoid to quinoid form, enabling more efficient hole transport. Recently, Teng et al. used the electronic effect of an interfacial coupling agent to treat PEDOT:PSS with trimethoxysilane, achieving the best PCE of 14.67% in Sn-PSCs [34] (Figure 5d).

**Figure 6:**
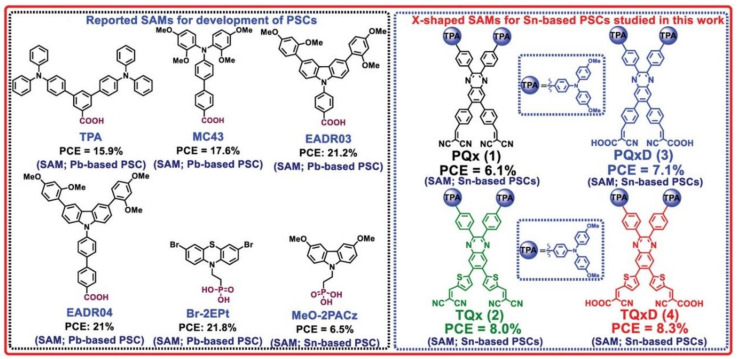
SAM molecules that have been reported. Right: Chemical structure of reported self-assembled monolayers (SAMs) for the PSCs. Chemical structures of SAMs: PQx (1), TQx, (2), PQxD (3) and TQxD (4) for Sn-based PSCs studied in the reference [56].

In recent years, self-assembled monolayers (SAMs) have garnered considerable attention as hole transport materials (HTMs) for inverted PSCs, aiming to overcome the issues associated with traditional HTLs [52]. However, fabricating high-quality tin perovskite films on hydrophobic HTM SAMs presents a challenge due to their poor wettability [53,54]. Improved film deposition methods and the design of new SAM molecules may be key to solving this problem [55,56]. For instance, Afraj et al. developed an X-shaped quinoline-based SAM that exhibited excellent hole extraction capabilities, resulting in Sn-PSCs with a PCE of 8.3% [56] (Figure 6).

**Figure 7:**
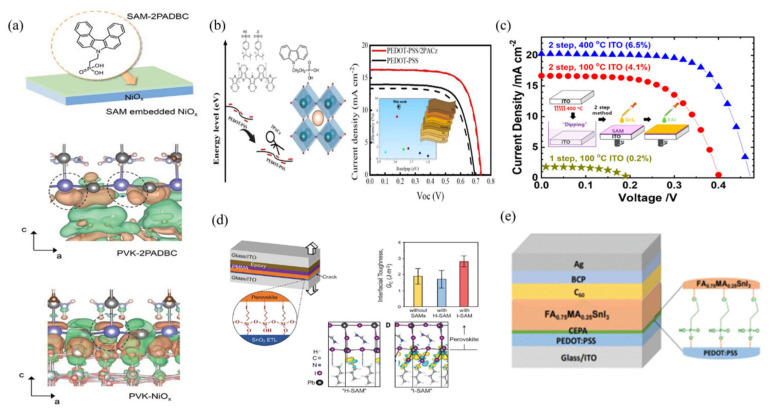
(**a**) Schematic diagram of SAM-2PADBC-embedded NiOx film and differential charge density simulation of the perovskite–NiOx and perovskite–2PADBC [57]. (**b**) The compact surface morphology of the Sn perovskite fabricated on bilayers consisting of 2PACz monolayer on PEDOT-PSS and the suppression of direct contact between PEDOT-PSS and perovskite film [59]. (**c**) In the two-step deposition, the SnI_2_ in DMSO and FAI in a cosolvent system were sequentially deposited using spin-coating to form FASnI_3_ [60]. (**d**) Mechanical behavior of the ETL/MHP interface [61]. (**e**) Schematic of the Sn-PSC based on CEPA buried interface modification [58].

Moreover, SAM molecules can be used to modify the surfaces of conventional HTLs. Recently, Li et al. introduced 2PADBC SAM at the tin perovskite/NiO_x_ interface to suppress interfacial redox reactions and reduce non-radiative recombination, achieving a PCE of 14.19% [57] (Figure 7a). Cao et al. designed a novel self-assembled molecule, 2-chloroethylphosphonic acid (CEPA), to modify the buried interface of Sn-PSCs [58]. The chemical anchoring of CEPA on PEDOT:PSS effectively reduced excess PSS and facilitated the formation of high-quality tin perovskite films, minimizing voids and impurity phases at the buried interface (Figure 7e). The reduced defects in the tin perovskite film, prepared on CEPA-modified PEDOT:PSS, significantly lowered the Urbach energy and suppressed non-radiative energy losses. As a result, the PCE of the CEPA-modified Sn-PSC based on FA_0.75_MA_0.25_SnI_3_ reached 10.65%, nearly 24% higher than that of the original perovskite device (8.57%). Similarly, Chen et al. reported high-performance tin-based PSCs with a wide bandgap of 1.62 eV and a PCE of 8.66%, using 2PACz SAM-modified PEDOT:PSS as the HTL [59] (Figure 7b). They also investigated the interaction between SAM anchoring groups (P–O−) and S^+^ in the interface, demonstrating how phosphonic acid deprotonation at the interface improved performance. However, the high hydrophobicity of carbazole-based SAMs may lead to uncontrolled crystallization of tin perovskites in SAMs. To address this, Song et al. developed a preheating process, using a two-step sequential deposition method to fabricate uniform tin-based perovskite layers [60] (Figure 7c). Thus, tailoring the functional group design to regulate crystallization and defect passivation is crucial for buried interface modification. For instance, halogen-terminated aliphatic SAMs have been effectively used in lead-based PSCs for buried interface modification [61] (Figure 7d). Their -I end group forms electrostatic bonds with the perovskite layer, and the shorter alkyl chain (*n* = 3) provides sufficient hydrophilicity for SAM coatings, promoting perovskite film deposition. Hence, functionalized aliphatic SAMs exhibit significant advantages in multifunctional buried interface modifications, particularly in crystallization control and defect passivation, contributing to improved tin-based PSC performance.

As the development of SAM materials progresses, more promising new materials may emerge. For instance, SAMs based on two-dimensional materials (e.g., graphene or transition metal sulfides), with their excellent charge transport properties and tunable optical characteristics, could become highly effective hole transport materials. The combination of these novel materials with conventional systems could lead to enhanced interfacial compatibility and improved device performance.

### 2.2. Polymer Materials in the Perovskite Precursor Solution

**Figure 8:**
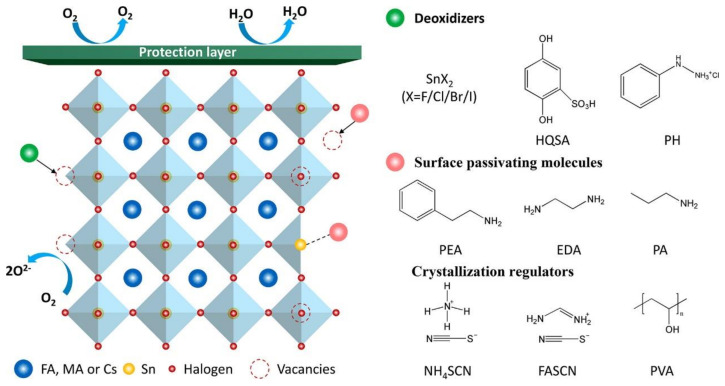
Schematic of the defects and functional molecules [63].

Additive engineering is widely recognized as an important means to improve the performance of tin-based perovskite solar cells (PSCs), primarily aimed at suppressing internal defects (such as tin vacancies and point defects) and external defects (such as grain boundary defects) [62,63] (Figure 8). The presence of internal defects significantly affects the optoelectronic properties of the material, while external defects can lead to decreased device stability. Therefore, the development of suitable additives is crucial for enhancing the quality of perovskite films. It is evident that the selection of additives not only impacts the fundamental properties of the materials but also directly relates to the practical application outcomes of the devices.

**Figure 9:**
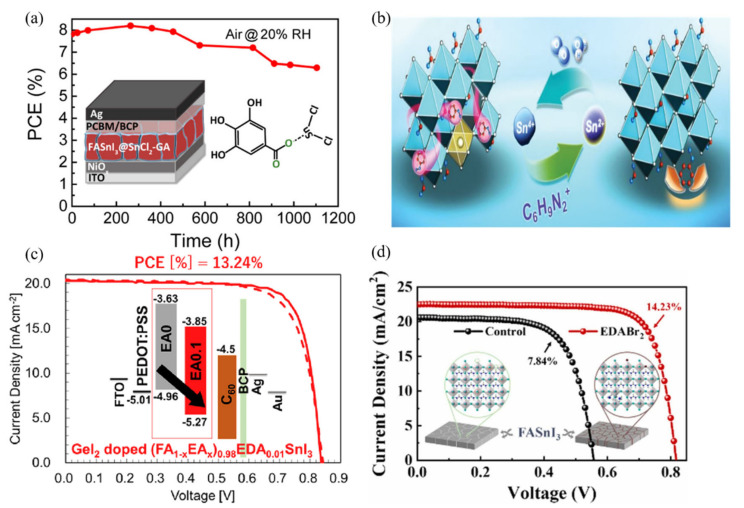
(**a**) A tin-based perovskite solar cell with significantly improved stability against oxidation was prepared by introducing hydroxybenzene sulfonic acid or a salt thereof as an antioxidant additive into the perovskite precursor solution [70]. (**b**) Schematic illustration of possible SRTSP mechanism for PHCl [73]. (**c**) Simultaneous band energy alignment and trap site passivation has been achieved by regulating the A-site cation of tin halide perovskites [71]. (**d**) Ethylenediammonium halide salts (i.e., EDAI2 and EDABr2) as additives in Sn perovskite [68].

In tin-based perovskite solar cells, the most commonly used additives are inorganic SnX_2_ compounds, such as SnF_2_ and SnCl_2_. These inorganic additives effectively suppress the oxidation of tin by altering the crystal structure and chemical environment of the perovskite film. With the continuous development of lead-based perovskites, an increasing number of organic additives have also been introduced into the precursor solutions for tin-based perovskites, enhancing the performance of the films. For instance, additives such as gallic acid (GA) (Figure 9a), phenylhydrazine hydrochloride (PHCl), ethylenediamine (EDA), phenethylammonium (PEA), and hypophosphorous acid (H_3_PO_2_) have been shown to promote the efficiency of tin-based devices [64,65,66,67,68,69,70,71,72,73,74]. These additives not only effectively prevent the oxidation of Sn^2+^ but also improve the crystallization quality of the films, thereby enhancing device performance. In fact, these additives optimize the microstructure, contributing to improved photoconversion efficiency and demonstrating significant potential in material design. Huang et al. found that the introduction of phenylhydrazine hydrochloride into FASnI_3_ perovskite films significantly reduced the generation of Sn^4+^ and inhibited the further degradation of FASnI_3_, ultimately achieving a power conversion efficiency (PCE) of 11.4% [73] (Figure 9b). This indicates that appropriate additives can effectively regulate the oxidation state of the material, thus improving its optoelectronic properties. Through this mechanism, researchers have illustrated the close relationship between the chemical stability of the material and the performance of the solar cells, providing important references for subsequent studies.

**Figure 10:**
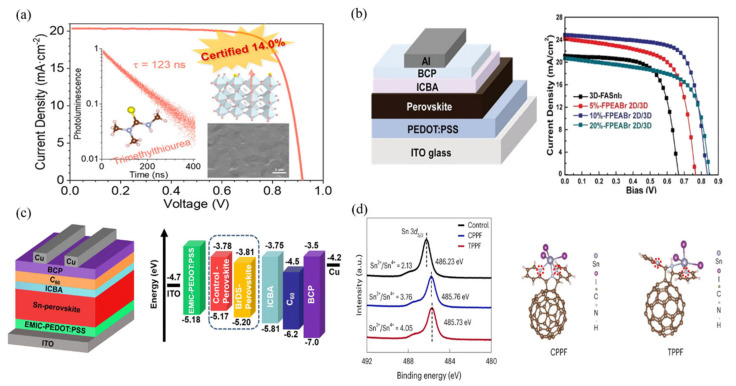
(**a**) Trimethylthiourea (3T) was introduced during spin-coating of FASnI_3_ films [75]. (**b**) Schematic device structure of the inverted perovskite devices. Current density–voltage (J-V) curves of the champion devices containing pure 3D FASnI_3_ and 2D/3D perovskite with different FPEABr [69]. (**c**) The structure of the Sn-based PSC device. The energy level diagram of the Sn-based PSC device [76]. (**d**) Sn 3d_5/2_ X-ray photoelectron spectroscopy spectra of the SnI_2_, CPPF-SnI_2_ and TPPF-SnI_2_ films. a.u., arbitrary units. Simulations of the interaction between the CPPF and perovskite components showing N (highlighted by red dashed circles) coordinating with one $SnI_{6}^{-}$ cluster and simulations of the interaction between TPPF and perovskite components showing N coordinating with two $SnI_{6}^{-}$ clusters [18].

Furthermore, the research by Shuzi Hayase et al. demonstrated that partially substituting ethylammonium cations for formamidinium cations could increase the efficiency of tin-based perovskite solar cells to 13.24% [71] (Figure 9c). This finding indicates that different combinations of cations can significantly affect the charge transport properties of the materials, optimizing cell performance. Zhao et al. further pointed out that the use of ethylenediamine halide salts (such as EDAI_2_ and EDABr_2_) as additives could enhance the PCE of the tin-based perovskite devices to 14.23% [68] (Figure 9d). This improvement may be attributed to the favorable interfacial structures formed by the additives in the films, which assist in enhancing charge transport efficiency. This illustrates the interrelationship between microstructural and macroscopic performance in material design, further advancing the field of renewable energy. Zhu et al. introduced trimethylthiourea (3T) in the spin-coating process of FASnI_3_ films (Figure 10a). This bifunctional ligand significantly improved the morphology and texture of FASnI_3_ films by spreading and connecting individual crystal particles. According to their findings, the carrier lifetime of the 3T-treated films reached a record 123 ns, and the open-circuit voltage was measured at 0.92 V, which is only 0.2 V lower than the theoretical limit for lead halide perovskites. These values indicate that the performance of the 3T-treated films approaches that of traditional lead-based perovskites [75]. A certified power conversion efficiency of 14.0% and excellent stability against humid air were also observed, marking these FASnI_3_ solar cells as the best in their category. Such experimental results demonstrate that the microstructure of the films and the selection of additives can significantly influence the electrical performance of the devices, promoting their feasibility in practical applications. He et al.’s research further indicated that replacing a portion of FAI with FPEABr in FASnI_3_ could yield an efficiency of 14.81% [69] (Figure 10b). This substitution not only improved the optoelectronic performance of the films but also potentially optimized the interfacial characteristics of the materials. Optimizing interfacial characteristics is another critical factor for enhancing device efficiency, especially in multilayer structures of solar cells.

Zhou et al. discovered that 2,8-dibromodibenzothiophene-S,S-dioxide (BrDS) is an ideal material for effectively reducing the high defect density in tin-based perovskite films [76] (Figure 10c). The strong interaction between the sulfone groups in BrDS and Sn^2+^ ions can effectively suppress the oxidation of Sn^2+^, promoting film stability. Furthermore, the incorporation of BrDS can compensate for the loss of iodide ions in the films by reducing crystal aggregation and phase separation, which is crucial for forming smooth, pinhole-free films that enhance overall device performance. This indicates that the multifaceted role of additives in improving film defects and stability is of profound significance for enhancing the performance of practical devices. Chen et al. synthesized two pyridine-substituted iron-rich porphyrins (PPF) with cis (CPPF) and trans (TPPF) configurations and utilized them as precursor additives [18] (Figure 10d). The spatial configurations of CPPF and TPPF significantly influence their electron density distribution and interaction with the perovskite components. Compared to CPPF, TPPF possesses spatially separated pyridine groups, which can capture more perovskite colloids through coordination bonds, thereby slowing down the crystallization process of the perovskite. The resulting perovskite films exhibited better crystal orientation and density. TPPF also resided at the grain boundaries, improving energy level alignment at the interface and inhibiting Sn^2+^ oxidation. Consequently, TPSC based on TPPF demonstrated a certified conversion efficiency of 15.14% and excellent stability, maintaining 99% and 93% of its initial efficiency after 3000 h of storage and 500 h of continuous illumination, respectively. This performance enhancement not only reflects the success of additive design but also provides new insights for future research.

The rational selection and regulation of additives play a crucial role in the development of tin-based perovskite solar cells. These studies not only offer new perspectives on understanding the functions of additives but also lay a solid foundation for the future industrial application of perovskite solar cells.

### 2.3. Polymer Materials on Top of Perovskite Films

**Figure 11:**
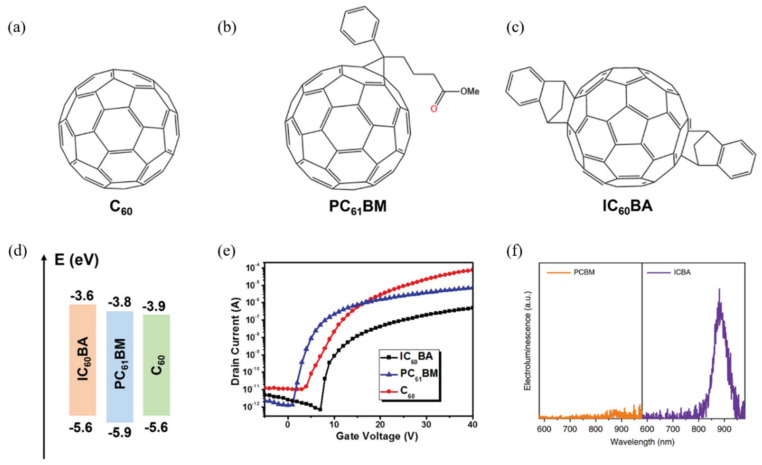
(**a**) C60. (**b**) PC61BM. (**c**) IC_60_BA. (**d**) Band structures of C_60_,PC_61_BM, and IC_60_BA. (**e**) The field effect transistor (FET) transfer characteristics of C_60_, PC_61_BM, and IC_60_BA. (**f**) Electroluminescence spectra of the Sn-based perovskites films with different ETLs (PC_61_BM and IC_60_BA) under a bias voltage of 2 V [38].

In lead-based perovskite solar cells, surface passivation of perovskite films is a crucial method for reducing defects, improving film quality, and enhancing device efficiency. In contrast, for tin-based perovskite solar cells, surface passivation primarily addresses the issue of energy level misalignment between the perovskite layer and the electron transport layer (ETL). For example, the energy level offset associated with C60 and [6]-phenyl C61-butyric acid methyl ester (PC_61_BM) [77,78] can lead to a reduction in the open-circuit voltage (Voc) of devices (Figure 11). Therefore, enhancing the LUMO level of the ETL can effectively increase the Voc of tin-based perovskite solar cells (TPSCs). Experimental evidence shows that the indene-C60 bisadduct (ICBA) can be utilized in tin-based perovskite solar cells [79,80,81], demonstrating better energy level alignment with tin-based perovskite, resulting in a larger Voc. Additionally, the shallow LUMO of ICBA hinders the injection of iodine ions, leading to a reduction in electron density and suppression of interfacial carrier recombination. In contrast, PC61BM promotes increased electron density due to long-range doping by iodine ions (ionic migration), accelerating interfacial carrier recombination with the p-type tin-based perovskite film. Consequently, the devices using ICBA exhibited a Voc output of 0.94 V and a power conversion efficiency (PCE) of 12.4%, while those using PC_61_BM showed only 0.60 V Voc and 7.7% PCE [82].

**Figure 12:**
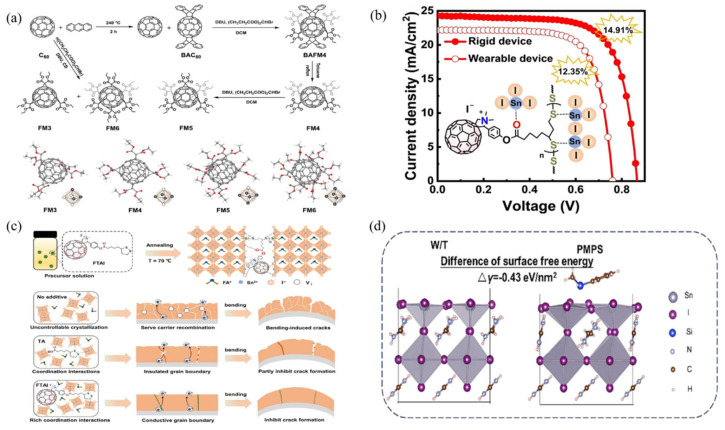
(**a**) Precise synthesis route of multidentate fullerenes, and single-crystal structures of FM3, FM4, FM5, and FM6, accompanied by corresponding fullerene structural models in the lower right corner; the solid vertex indicates that the position is occupied by a functional group, while a hollow vertex indicates that it is not occupied [83]. (**b**) Design and synthesis of a cross-linkable fullerene, lipoic acid-functionalized C60 fuller pyrrolidinium iodide (FTAI) for the preparation of high-efficiency devices [84]. (**c**) Schematic illustrations of the effect of FTAI on perovskite film. Molecular structure of FTAI and diagram of cross-linked FTAI interact with perovskite. Schematic diagrams of formation and bending performance of the control and TA- and FTAI-based perovskite films [84]. (**d**) Optimized slab model (1 0 0) of perovskite of W/T and PMPS [85].

Recently, a growing number of custom-designed materials have been employed for interfacial passivation of tin-based perovskite films. Sun et al. synthesized four well-defined multidentate fullerene molecules, labeled FM3, FM4, FM5, and FM6, each containing three, four, five, and six diethyl malonate groups, to be used as interfacial layers in TPSCs [83] (Figure 12a). It was observed that increasing the number of functional groups on these fullerenes leads to a shallower LUMO energy level and enhances the interfacial chemical interactions. Among these, FM5 exhibited suitable energy levels and strong interactions with perovskite, effectively enhancing electron extraction and defect passivation. Furthermore, the unique molecular structure of FM5 allows for tight stacking of the exposed carbon cage with the upper fullerene cage upon interaction with perovskite, facilitating efficient charge transfer and protecting the perovskite from moisture and oxygen damage. As a result, devices based on FM5 achieved a champion efficiency of 15.05%. Hou et al. designed and synthesized a cross-linkable fullerene, thiol-functionalized C60 fulleropyrrolidine iodide (FTAI) [84], which interacts with the perovskite components to finely tune the crystallinity of the perovskite films (Figure 12b). The resulting perovskite films displayed larger grain sizes and better energy level alignment with the electron transport material, effectively improving carrier extraction efficiency (Figure 12c). The rigid devices based on FTAI achieved a champion efficiency of 14.91% and enhanced stability. In addition to this, the development of new methods is also a strategy. Zhang et al. developed a strategy using polysilanes, particularly polymethylphenylsilane (PMPS) and diphenylpentasilane (DPPS), to improve the quality of tin-based perovskites [85] (Figure 12d). Surface passivation with PMPS led to a PCE of 14.18% and better stability. Further characterization indicated that PMPS serves a dual role: it provides a smooth surface morphology and enlarged grain sizes (enhancing short-circuit current, Jsc) while also acting as a reducing agent for Sn^4+^ and a surface energy level regulator (enhancing Voc).

**Figure 13:**
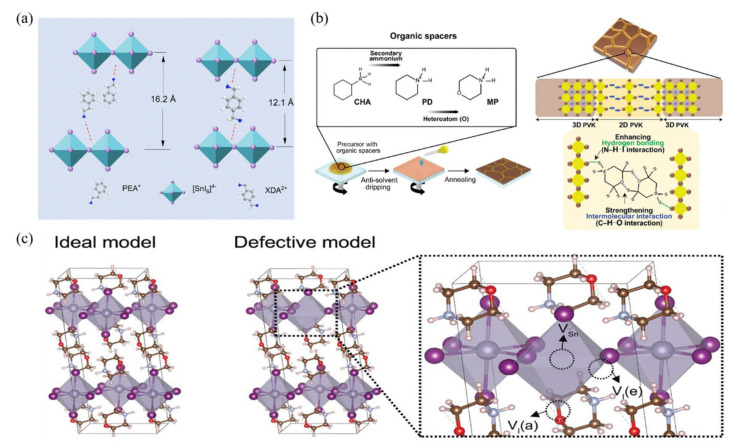
(**a**) Schematic of interaction between [SnI_6_]^4−^ and PEA (left) or XDA (right) [86]. (**b**) Left: Deposition process of 2D/3D perovskite using different organic spacers; cyclohexylammonium (CHA), piperidinium (PD), and morpholinium (MP). Right: Schematic illustration of the crystal structure of MP-based 2D/3D perovskite [87]. (**c**) Structural model of MP-based 2D perovskite. Structural model of 2D MP-based perovskite with vacancies. V_I_(e): neutral I vacancies in the equatorial position; V_I_(a): neutral I vacancies in the apical position; V_Sn_: neutral Sn vacancies [87].

The growth of a 2D film on perovskite films to form a 2D/3D structured perovskite is also an effective passivation strategy. Zang et al. constructed quasi-2D tin-based perovskite films using a mixture of monoammonium and diammonium terminal ligands [86] (Figure 13a). The use of diammonium ligands reduced the total number of terminal ligands in the films and delayed the growth of the perovskite, enhancing film orientation. These improvements increased carrier mobility and diffusion lengths, resulting in a 14.3% increase in current density and power conversion efficiency. Cui et al. reported a novel cyclic organic spacer, morpholinium iodide (MPI), for developing structurally stable 2D/3D perovskites [87] (Figure 13b). The introduction of secondary ammonium and ether groups in the cyclic spacer enhances its rigidity, leading to an increase in hydrogen bonding and intermolecular interactions within the 2D perovskite. These enhanced interactions facilitate the formation of highly ordered 2D/3D perovskites with low structural disorder, effectively passivating Sn and I defects (Figure 13c). Consequently, perovskite solar cells based on MPI achieved a PCE of 12.04%, demonstrating excellent operational stability and oxidative stability.

Additionally, there have been attempts to incorporate materials previously used in lead-based devices into tin-based devices. Recently, Chan et al. applied the commonly used ligand isobutylammonium iodide (iso-BAI) for surface treatment of tin-based perovskite [88]. Unlike the passivation effects observed in lead-based perovskites, this treatment led to surface recrystallization driven by the higher solubility of tin-based perovskite in common solvents. By carefully designing the solvent composition, effective modification of the perovskite surface was achieved while maintaining the integrity of the bulk material. This treatment improved surface crystallinity, reduced surface strain and defects, and enhanced charge transport.

Overall, these advancements in materials and strategies provide new solutions for enhancing the performance of tin-based perovskite solar cells and suggest promising avenues for future research in perovskite material development. With continuous exploration of novel materials, the potential for higher efficiency and improved stability in perovskite solar cells appears increasingly attainable.

## 3. Discussion

Previous studies have primarily focused on optimizing PEDOT:PSS to improve the buried interface, with numerous measures implemented in this regard. However, in recent years, the rapid development of self-assembled materials in lead-based perovskite solar cells has prompted initial attempts to apply self-assembled materials in tin-based perovskite solar cells. The surface chemical properties of self-assembled materials can be precisely tuned through the selection and arrangement of molecules, allowing for the design of interfaces suitable for perovskites, thereby enhancing the separation efficiency of electrons and holes. Additionally, a protective film can be formed at the interface, reducing the erosion of the perovskite layer by external factors such as moisture and oxygen, thus improving device stability and lifespan.

When designing novel self-assembled molecules, two main aspects should be considered. Firstly, anchoring to the transport layer can be achieved by incorporating different functional groups, such as carboxylic acids or amines, into the self-assembled molecules. These groups can chemically bond with the perovskite layer or PEDOT, enhancing interfacial adhesion and stability. Understanding the self-assembly mechanisms of these molecules (such as hydrogen bonding and hydrophobic interactions) can also aid in predicting their behavior during interface formation, facilitating better design. Secondly, modifications to the buried interface of the perovskite layer can improve charge separation and transport efficiency in tin-based perovskites, contributing to enhanced overall cell performance. The introduction of self-assembled materials not only protects the perovskite layer but also enhances its adaptability to environmental changes, thereby extending the device’s operational lifespan.

In tin-based perovskite solar cells, optimizing the perovskite precursor solution is a significant research focus. This involves compositional tuning of the A, B, and X ions in the tin-based perovskite ABX_3_ structure. Specifically, when adjusting the A-site ions, the use of macromolecular materials for dimensional control has emerged as an effective strategy to enhance device efficiency and stability.

Additive modulation represents another crucial method for optimizing both the perovskite precursor solution and the films. A variety of organic and inorganic materials have been incorporated into the perovskite precursor solution. In addition to traditional inorganic materials, an increasing number of researchers are exploring new organic materials for inclusion in the precursor solution. These new materials can prevent oxidation of the precursor solution and, when formed into films, can reduce defects in the active layer, thereby improving film quality.

Regarding the identification of suitable additive materials, existing research indicates that fullerene materials show considerable promise. Direct use of fullerene materials as additives is a viable research direction, with experiments aimed at finding the most effective additives. Moreover, developing new materials based on fullerene structures may become a future research hotspot. By introducing targeted functional groups to proven fullerene materials, it may be possible to further enhance the resistance to oxidation of divalent tin and improve the film quality of the perovskite layers.

Finally, we present a table summarizing the device efficiencies and electrical parameters of devices fabricated using self-assembled molecules and fullerene materials, as shown in Table 1. Most of the devices achieve efficiencies above 12%, indicating the effectiveness of the materials used.

## 4. Conclusions

This paper summarizes the various materials recently employed in tin-based perovskite solar cells, focusing on their roles at the buried interface, within the active layer, and on the surface of the perovskite layer. Notably, self-assembled molecules and fullerene materials have shown great potential. The discussion highlights the functions of these materials within the devices and provides insights into their distinct contributions. Furthermore, suggestions are offered on how to design and utilize new materials in future research.

## Figures and Tables

**Table 1:** The device efficiencies and electrical parameters of devices fabricated using self-assembled molecules and fullerene materials.

Materials	Voc (V)	Jsc (mA/cm^2^)	FF (%)	PCE (%)	Ref.
Aromatic Diammonium Acetate Salts	0.826	20.7	70.8	12.1	[45]
Trimethoxy (3,3,3-Trifluoropropyl)-silane	0.910	22.6	70.4	14.36	[34]
X-shaped quinoxaline-based organic dyes	0.574	21.1	68.8	8.32	[56]
(4-(7*H*-dibenzo [*c*,*g*]carbazol-7-yl)ethyl)phosphonic acid	0.825	23.3	74.0	14.19	[57]
2-chloroethylphosphonic acid	0.640	23.2	71.8	10.65	[58]
[2-(9H-carbazol-9-yl)ethyl]phosphonic acid	0.740	16.2	73.0	8.66	[59]
Ethylenediammonium halide salts	0.817	22.5	77.4	14.23	[68]
Trimethylthiourea	0.920	20.4	76.7	14.30	[75]
2,8-dibromo-dibenzothiophene-*S*,*S*-dioxide	0.790	23.9	79.5	14.98	[76]
Pyridyl-substituted fulleropyrrolidines	0.856	24.8	72.4	15.38	[18]
[6,6]-phenyl-C61-butyric acid methyl ester	0.949	17.4	74.9	12.42	[82]
Multidentate fullerene molecules with 3, 4, 5, and 6 diethylmalonate groups	0.860	24.5	71.1	15.05	[83]
Thioctic acid functionalized C_60_ fulleropyrrolidinium iodide	0.866	24.6	70.0	14.91	[84]
Polymethyl-phenyl-silane	0.820	24.3	71.0	14.18	[85]
Mixed monoammonium and diammonium terminal ligands	0.900	20.7	76.9	14.31	[86]
Morpholinium iodide	0.803	20.6	73.1	12.04	[87]
Iso-butylammonium iodide	0.719	26.1	75.7	14.20	[88]

## Data Availability

The raw data can be obtained from the corresponding authors upon reasonable request.
